# Towards Predictive Communication: The Fusion of Large Language Models and Brain–Computer Interface

**DOI:** 10.3390/s25133987

**Published:** 2025-06-26

**Authors:** Andrea Carìa

**Affiliations:** Department of Psychology and Cognitive Science, University of Trento, 38068 Rovereto, Italy; andrea.caria@unitn.it

**Keywords:** brain–computer interface, EEG, electroencephalography, human–computer interaction (HCI), human–machine interaction (HMI), deep learning, large language models (LLMs), predictive writing, transformer models, BCI spellers

## Abstract

Integration of advanced artificial intelligence with neurotechnology offers transformative potential for assistive communication. This perspective article examines the emerging convergence between non-invasive brain–computer interface (BCI) spellers and large language models (LLMs), with a focus on predictive communication for individuals with motor or language impairments. First, I will review the evolution of language models—from early rule-based systems to contemporary deep learning architectures—and their role in enhancing predictive writing. Second, I will survey existing implementations of BCI spellers that incorporate language modeling and highlight recent pilot studies exploring the integration of LLMs into BCI. Third, I will examine how, despite advancements in typing speed, accuracy, and user adaptability, the fusion of LLMs and BCI spellers still faces key challenges such as real-time processing, robustness to noise, and the integration of neural decoding outputs with probabilistic language generation frameworks. Finally, I will discuss how fully integrating LLMs with BCI technology could substantially improve the speed and usability of BCI-mediated communication, offering a path toward more intuitive, adaptive, and effective neurotechnological solutions for both clinical and non-clinical users.

## 1. Introduction

Brain–computer interfaces (BCIs) are neurotechnologies that enable real-time acquisition and decoding of brain activity for the control of external devices and communication systems [1,2]. In individuals with severe neurological disorders or brain injuries affecting neuromuscular pathways, BCIs offer a non-muscular communication channel, allowing users to convey intentions and interact with their environment despite profound physical impairments [3,4,5,6].

Invasive BCIs, including intracortical neuroprostheses, have demonstrated high-performance speech decoding and text generation in individuals with paralysis [7,8,9]. Recent advances include systems capable of translating imagined handwriting into real-time text at speeds up to 90 characters per minute using recurrent neural networks (RNNs) with integrated language modeling and autocorrection [8,10]. Other approaches have enabled the synthesis of speech from silent articulation-related neural activity recorded via high-density electrocorticography, with outputs rendered in real time through audiovisual avatars [11]. While these invasive systems currently outperform standard augmentative and alternative communication (AAC) technologies [12,13], their clinical translation remains limited by medical risk, signal degradation, and the logistical complexities of surgical implantation.

Non-invasive BCIs, typically based on electroencephalography (EEG), present a more practical and scalable alternative. Although current BCI spellers’ systems have not yet achieved direct speech decoding [14,15,16], they are effective for enabling communication in individuals with minimal residual motor function. EEG-based BCI spellers do not decode speech-related brain activity per se [17] but instead leverage neural signals associated with visual, motor, or cognitive events to drive user interfaces. Common paradigms exploited visual evoked potentials (VEPs), the P300 response, and sensorimotor event-related desynchronization/synchronization patterns to identify user intent through attention to target visual stimuli. BCI-mediated typing via visual interfaces typically requires users to focus on letters or words rapidly displayed in various visual configurations. Detection of specific EEG components relies on pre-trained classifiers that infer the intended character or word in relation to the modulation of selected EEG components.

EEG-based BCI spellers often involve trade-offs between speed, accuracy, and usability; higher accuracy may require repetitive, attentionally demanding stimulation, which can hinder user experience and system adoption. Despite these limitations, recent work has demonstrated that high-performance EEG-BCI spellers are feasible [1,18,19]. For example, event-locked steady-state visual evoked potential (SSVEP)-based BCI systems have achieved information transfer rates (ITRs) exceeding 5 bits/s (~12 words per minute) using frequency-phase modulation and user-specific decoding [18].

Moreover, deep learning-based classifiers, using convolutional neural networks (CNNs), applied to SSVEP signals have further improved performance, enabling synchronous communication with an average ITR of 701 bits/min, and asynchronous with an ITR of 175 bits/min (an average of 35 error-free letters per minute) [19,20]. While impressive and promising, from an end-user perspective, SSVEP-BCI spellers are visually demanding and potentially unsuitable for users with conditions like photosensitive epilepsy. As such, enhancing interface usability and communication efficiency, rather than focusing solely on signal decoding, is a priority for next-generation BCI spellers’ design.

To address these challenges, integrating intelligent predictive systems, particularly natural language models, into BCI spellers has attracted increasing interest. Even early integrations of simple statistical language models into BCI communication systems have led to measurable improvements in speed (resulting on average in a ~30% bit-rate increase), accuracy, and interface usability [21,22,23]. These systems support word completion, word suggestion, dynamic stimulus adjustment, unsupervised learning, and error correction, contributing to more fluid communication [21]. Despite these gains, traditional models are limited in their ability to incorporate semantic context or model complex linguistic patterns.

Recent advances in artificial intelligence, particularly the emergence of large language models (LLMs) that have revolutionized natural language processing (NLP) by capturing complex linguistic patterns, offer new opportunities to transform BCI-mediated communication. Trained on massive text corpora, LLMs such as GPT, Transformer-XL, and Reformer demonstrate human-like language understanding and generation capabilities by modeling multifaceted syntactic and semantic relationships [24,25,26]. While their integration with BCIs is still nascent, early studies suggest the LLMs’ potential to substantially enhance non-invasive communication systems, both in terms of predictive accuracy and typing efficiency [27,28,29].

Building on these developments, this perspective article provides an overview of the current state and future potential of integrating cutting-edge LLMs with non-invasive BCI spellers. I will first trace the evolution of language models from traditional NLP tools to state-of-the-art LLMs and review their application in predictive writing systems. Next, I will summarize previous efforts to combine language modeling with BCI spellers’ implementations and highlight recent pilot studies exploring LLM–BCI integration. I will then examine how, despite advancements in typing speed, accuracy, and user adaptability, the fusion of LLMs and BCI spellers still faces key challenges such as real-time processing, robustness to noise, and the integration of neural decoding outputs with probabilistic language generation frameworks. Finally, I will discuss how fully integrating LLMs with BCI technology could substantially improve the speed and usability of BCI-mediated communication, offering a path toward more intuitive, adaptive, and effective neurotechnological solutions for both clinical and non-clinical users.

## 2. Predictive Writing: Intelligent Text Entry Systems

The introduction of intelligent text entry systems made human–computer interaction faster and more efficient [30]. Intelligent typing systems are now ubiquitous on mobile devices such as smartphones and tablets as well as in desktop environments. Intelligent typing systems rely on predictive algorithms that significantly reduce users’ typing effort and time by supporting word completion and offering word suggestions. Based on predefined language models, these systems provide shortcuts for entering words or phrases that are most predictable in a given context.

Language models are computational tools designed to enhance performance in natural language processing tasks, including both the understanding and generation of human language. A fundamental feature of most modern language models is autoregression or probabilistic text generation, the ability to predict the likelihood of word sequences or generate text based on an input prompt. This is made possible by specific neural network architectures designed for processing sequential data.

The integration of predictive language modeling into writing interfaces was initially proposed to support text input for individuals with motor impairments or limited typing capabilities [30]. More recently, predictive language models have been developed for broader applications, offering benefits such as reduced motor effort, fewer typing errors, and improved writing assistance for all users. These predictive writing approaches, whether based on statistical methods [24,25,26] or other AI techniques, have supported, with varying degrees of effectiveness, word completion, word suggestions, and automatic error correction. To date, predictive text entry systems built on different language models have shown heterogeneous effects on typing speed, accuracy, and suggestion usage [31,32].

### 2.1. Early Language Models in Predictive Writing

One of the earliest predictive systems employing statistical methods [33] was based on the Markov chain approach and was applied in the contexts of NLP and entertainment computing. The Markov chain language model attempted to mimic human language by generating next-word suggestions based on their probability in a text *corpus* related to a given topic. Other statistical approaches relied on word frequency or word sequence frequencies within a given context.

The simplest word frequency-based language model is the Unigram model, where the probability of a token (a basic unit of text, typically a word, subword, or character) is calculated independently of any preceding tokens or context. Extensions of the Unigram model led to the development of Bigram, Trigram, and more generally N-gram probabilistic models, where the probability of the next word depends on the previous 1, 2, or N−1 words, respectively. In the case of N-gram models, probabilities are assigned to entire sequences [34]. In general, N-gram models [35] exhibit limitations when encountering unseen words or complex linguistic phenomena. They are also prone to overfitting, struggling to generalize beyond their training data, which makes them less adaptable in diverse contexts.

More advanced predictive language models have been developed using artificial neural networks such as CNNs and recurrent neural networks (RNNs) [36]. RNNs are designed for sequence-based data as they, unlike traditional feedforward neural networks, possess a built-in memory that allows them to retain information about previous inputs. This makes them particularly well-suited for tasks where context matters, such as time-series prediction, NLP, and speech recognition [37]. However, RNNs have practical limitations with long sequences due to issues such as the vanishing gradient problem—where the influence of earlier inputs fades exponentially over time. RNN-based next-word prediction typically relies on preserving information from previous words through the hidden states of the network’s hidden layers. A specialized class of RNNs, known as long short-term memory (LSTM) networks, has shown remarkable effectiveness in speech recognition and language modeling, significantly outperforming traditional models [38].

Modern predictive systems can employ a range of approaches to determine the next character, word, or phrase to be entered. These may include language models trained on large-scale text corpora from diverse sources [39,40], user-specific predictions based on prior writing history, input from conversation partners [41], or hybrid strategies that combine multiple methods. Some implementations have also embedded contextual phrase previews [31], with complete-sentence replies [42], or proposed a single highly probable phrase continuation [43]. Collectively, these predictive typing systems effectively support users with a wide range of typing abilities, including those with motor impairments or learning disabilities such as dyslexia or dysgraphia.

### 2.2. Large Language Models in Predictive Writing

Nowadays, all the above-mentioned models have been largely superseded by LLMs, which primarily consist of large-scale pretrained autoregressive models that have significantly improved performance in several NLP tasks. In contrast to earlier models, which were typically capable of solving only specific tasks, LLMs can be applied across a wide range of diverse scenarios, with exceptional learning capabilities [44]. LLMs mainly consist of deep learning algorithms composed of multiple neural network layers [45]. These models are trained on massive datasets to acquire a large collection of language features and statistical information regarding linguistic relationships [46]. LLMs are generally built on the transformer neural network architecture [47], which can be used for various NLP tasks, including predictive text generation based on user input, as well as generating responses based on provided context or instructions to simulate human conversational behavior. Transformer models use the self-attention mechanism, dynamically weighing different parts of the input sequence, to process entire sequences at once, rather than one step at a time. The transformer architecture, by supporting parallelization, allows for the efficient capture of long-range dependencies in text, outperforming previous models. Key differences between autoregressive and transformer-based models are summarized in Table 1. Examples of popular LLM include Google’s Pathways Language Model (PaLM and PaLM2), T5 or Text-to-Text Transfer Transformer, the Bidirectional Encoder Representations from Transformers (BERT), the Robustly Optimized BERT Pretraining Approach (RoBERTa), the lite Bert variant (ALBERT), XLNet, Transformer-XL and the Generative Pre-trained Transformer (GPT), and DeepSeek LLM.

Most modern LLMs, including the GPT series, leverage parallel processing of multiple text sequences for more efficient training and inference compared to previous RNN-based models. In addition, LLMs can feature qqyuan@sgg.whu.edu.cn, an emergent capability that enables these models to adapt to new tasks or information based on examples or context provided in the input prompt, without the need for fine-tuning of parameters. This capability gives LLMs the flexibility to adapt to diverse scenarios. LLMs can also incorporate reinforcement learning from human feedback (RLHF), which allows for the fine-tuning of models based on human input and preferences. In RLHF, mode outputs are evaluated by humans (e.g., during question–answer interactions), with preferred responses being reinforced during the training process.

In general, the performance of LLMs can be assessed either through subjective human evaluation or more objectively through standard linguistic metrics. Human evaluation of LLMs’ capabilities on general natural language tasks is efficient in real-world applications, as it provides a more comprehensive and accurate assessment. In contrast, existing automatic evaluation tools can offer standard metrics of model performance without requiring intensive human supervision. Typically, automatic evaluation tools allow for the assessment of accuracy (how precise a model is on a given task), calibration (how well the model’s confidence scores align with the actual correctness of its predictions), robustness (the ability to maintain consistent and accurate performance under varying inputs, including adversarial perturbations, out-of-distribution data, and noisy or ambiguous data), and efficiency. Accuracy in LLMs can be measured using various metrics such as Exact Match (the percentage of predictions that exactly match the ground truth—target or reference—without any differences in words, order, or punctuation), F1 score (which combines both precision—how much of the generated text is relevant—and recall—how much of the reference text is captured—to produce a balanced score), BLEU (which compares how many words from the predicted sentence—candidate—are also present in the ground truth sentence by evaluating n-grams), ROUGE (which extends on BLEU and includes both precision and recall terms by calculating the F1 score), and Meteor (MT) (which calculates both precision and recall) scores.

In this regard, a survey examining studies assessing the performance of various LLMs (including ChatGPT(GPT-1), GPT-3.5, GPT-4, PaLM, and LLaMA) showed that current autoregressive models exhibit unprecedented performance in natural language understanding and generation [48]. For instance, with respect to text generation, the large-scale pretrained ChatGPT model demonstrated the best overall performance in English (PersonaChat, DailyDialog, EmpatheticDialogue) and Chinese dialogue (LCCC) generation, as measured by several metrics including BLEU and MT (B-4 = 0.52, MT = 9.78 on PersonaChat; B-4 = 0.56, MT = 10.13 on DailyDialog) when compared to other LLMs such as Open-LLaMA (B-4 = 0.00, MT = 5.86 on PersonaChat; B-4 = 0.46, MT = 5.94 on DailyDialog) and Flan-T5-XXL (B-4 = 0.43, MT = 6.15 on PersonaChat; B-4 = 0.42, MT = 6.64 on DailyDialog) [49].

Overall, LLMs perform well in generating text, producing fluent and precise linguistic expressions. They also show impressive performance in tasks involving language understanding, including sentiment analysis (assessing and identifying the emotional connotation of the text) [50], and, more broadly, in text classification. However, LLMs tend to show modest or poor performance in tasks such as natural language inference (determining whether a given premise leads to a true, false, or undetermined hypothesis), discerning semantic similarity (measuring how closely related two pieces of text are in meaning), abstract reasoning (the ability to generalize, infer patterns, and apply logical thinking beyond simple pattern recognition or memorization), and complex contexts interpretation [48]. Current LLMs have limitations in certain aspects of language processing, particularly in more complex and ambiguous contexts, and face challenges in integrating real-time or dynamic information, making them less effective in tasks that require fast adaptation to changing scenarios [48].

In short, these findings suggest that BCI-mediated communication could be significantly enhanced by transitioning from early, simple probabilistic language models to more advanced models leveraging deep learning and transformer architecture, such as LLMs. Indeed, these models can improve text generation and understanding in BCI applications and overcome previous models by providing advanced features such as fluent text production, sentiment analysis, text classification, and question–answering. However, further research is needed to address current limitations, such a resolving semantic similarity, enhancing abstract reasoning and natural language inference, and enabling real-time adaptation, in order to ultimately facilitate more complex human–computer interactions.

## 3. Language Models and Brain–Computer Interfaces

An increasing number of studies indicate that the incorporation of predictive algorithms, exploiting the statistical structure of language [33,51], into BCI spellers leads to improved writing performance in both healthy individuals and patients [21,22,52,53].

### 3.1. Integration of Early Language Models with BCI Spellers

The first attempts to integrate language models into BCI involved combining a predictive spelling program (e.g., Quillsoft WordQ2) [54] and customized predictive text entry software [55] with a P300-based Matrix Speller. Early implementations often used a two-stage letter/word selection process, both stages relying on random flashing of rows and columns in a matrix that contained letters and numbers. In the first stage, a few initial letters were selected to form a prefix of predefined length. This prefix was then used to generate a fixed number of word suggestions based on the language model’s prediction. In the second stage, the user selected the intended word either by focusing on the number associated with the word (presented in a numeric matrix) [54] or by selecting the word directly from an extended matrix that included the suggested words [55].

Further systems were proposed, such as those combining the matrix speller with a built-in dictionary that predicted the most likely words based on a few selected characters [56]. Moreover, BCI matrix spellers adopted the T9 (text on nine keys) predictive text technology, originally used on mobile phones with numeric keypads. T9 used a built-in dictionary to predict the most likely word from a sequence of keypresses. If multiple words matched the same key sequence, users could cycle through alternatives. Notably, the system learned from user behavior over time, enhancing prediction accuracy. As in earlier systems, these implementations included a word prediction module and used a two-stage word selection process.

Furthermore, various previous studies explored integrating N-gram language models, ranging from naïve Bayes models to partially observable Markov decision processes and hidden Markov models [57,58,59,60]. These efforts resulted in a significant reduction in typing time and in the generation of more user-friendly interfaces by outputting complete words from only a few initial character inputs. For instance, using a modified T9 interface, the P300-BCI system achieved an average typing time per word of 1.67 min, compared to 3.47 min with a conventional speller, a 51.87% improvement over a conventional spelling system [61,62].

However, most of these models were unable to consider semantic context. As a result, their natural language representations were limited, leading to potentially accurate character prediction but lower prior probability assignments to the actual target characters. Due to limited character history, high probability might be assigned to strings that locally resemble correct patterns but lack contextual meaning.

So far, BCI studies exploiting language models have demonstrated improved typing speed via word prediction, word completion, and error correction [54,55,56,62] and enhanced character classification. This latter has been achieved, for instance, through model-derived priors in classification algorithms that reduce the space of likely character sequences [23,63]. In particular, probability distributions of automaton-generated words (a probabilistic language model), derived from a particle filtering algorithm, were used as priors to classify EEG signals in a P300 speller system using Bayesian inference [23]. The online performance of particle filtering classifier significantly outperformed the Hidden Markov Model (HMM)-based approach, achieving an average selection rate of 8.64 characters/min (HMM = 8.05), accuracy of 89.70% (HMM = 83.74), and bit rate of 37.31 bits/min (HMM = 30.69). This enhanced BCI system also improved typing performance in ALS patients, reaching up to 84% accuracy in online spelling sessions [52]. These results suggest that integrating context-aware language models into BCI classification can substantially benefit system performance.

Additionally, other studies have explored the integration of more advanced language models to further boost BCI performance [64,65]. One early attempt to include semantic context proposed a joint word–character finite-state approach that combined word-level and character-level language models to enhance letter prediction. This mixed-context model outperformed a purely character-level model in noisy input conditions [65].

Notably, BCI classification of EEG signals can produce erroneous output that does not reflect the user’s intention. This misclassification introduces noise into the system, which can hinder the performance of language models typically trained on clean text. These models generate predictions based on the assumed accurate input and may struggle with the noisy outputs from BCIs. To address this, language models can be trained on text containing character-level noise and configured to process multiple candidate histories, instead of a single token sequence of token [66,67]. In this direction, Dudy and colleagues considered ambiguous BCI signal outputs classification and allowed the language model to “interpolate” the noisy input to predict the next letter. However, this approach did not yield significant performance improvements in a word–character hybrid model [65].

More recently, an RNN-based language model was implemented for online predictive typing using noisy histories [64]. This model, based on long short-term memory networks capable of capturing long-term dependencies, was trained on synthetic noisy data. It outperformed N-gram models trained on either noisy or clean text and demonstrated improved generalizability in predictive typing tasks. Furthermore, the authors reported that including multiple candidate histories can enhance predictive performance [64]. However, the model required substantial training data, making BCI systems based on it more complex and less flexible.

### 3.2. Integration of Large Language Models with BCI Spellers

The rapid advancement of LLMs promises to drive further advancements in BCI systems, particularly in the domain of assistive communication. Recent models such as Reformer [68], Transformer-XL [69], and lightweight versions of GPT (Turbo variants) [45,70] are sufficiently compact to be feasibly integrated into BCI systems. However, despite their potential, clear demonstrations of combining LLMs with BCI spellers to support real-time communication in both healthy individuals and clinical populations have yet to be reported.

**Figure 1:**
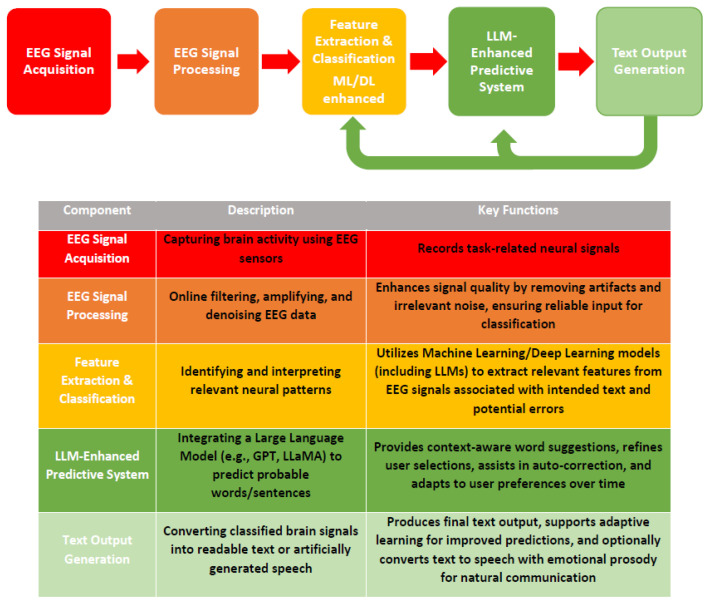
Schematic representation of a future prototypic BCI speller integrating LLM. An LLM-BCI speller consists of the following key components: 1. EEG Signal Acquisition; 2. EEG Signal Processing, Feature Extraction and Classification (currently, LLMs are not commonly employed for EEG feature extraction; however, future implementations may integrate these models into neural decoding pipelines, see Section 4.1.3); 3. LLM-Enhanced Predictive System; 4. Text Output Generation. This integration is anticipated to enhance typing speed, accuracy, and usability.

Nonetheless, some pilot studies have explored the prospective integration of various LLMs [27,28] into both human–computer interaction (HCI) and BCI systems (see Figure 1). A recent study proposed an LLM-powered user interface to enhance text entry in AAC. The system, named SpeakFaster, accelerated communication for non-AAC participants by reducing motor actions by 57% and also improved performance in two eye-gaze AAC users with amyotrophic lateral sclerosis (ALS) [28].

An exploratory study assessing how different LLMs might improve predictive typing with BCI demonstrated particularly impressive performance by GPT-2, which outperformed a unigram-based model [27]. The authors examined the potential of GPT, GPT-2, and Transformer-XL to boost predictive typing with BCI. However, the evaluation of character-level prediction was conducted using two existing datasets—the ALS PhraseSet and the Switchboard corpus—rather than in real-world BCI scenarios. Model performance was assessed using metrics well-suited to sequential character presentation, and thus, considered relevant for BCI systems, such as Mean Reciprocal Rank (MRR) and Recall at rank k (Recall@k). MRR computes the average over different predictions of the reciprocal rank of the correct target letter if it appears in the top k suggestions, or zero otherwise. Recall@k reflects the proportion of instances in which the correct character was included among the top k predictions. The study also provided insight into how different LLM architectures generate character-level predictions. The Reformer model was used to generate a probabilistic distribution over its character vocabulary for next-character prediction, which served as the final outcome. For the Transformer-XL model, character-level predictions were produced by first identifying words from a fixed vocabulary whose prefixes matched the last partially typed word in the input. The model then renormalized the probability distribution over these words and marginalized over the first character following the matched prefix to obtain the next-character distribution.

In the case of GPT and GPT-2 models, byte-pair encoding (BPE) was used for tokenization, generating a fixed-size vocabulary composed of common English subword units. Following a similar strategy to that of Transformer-XL, the models predicted the last subword unit over the entire vocabulary. After selecting subword units with prefixes matching the partially typed final subword, renormalization and marginalization yielded a character-level probability distribution. Additionally, a Beam Search algorithm—a heuristic method that explores the most promising nodes in a limited search space—was applied to generate multiple subword predictions without constraining potential continuations.

Preliminary findings indicated that GPT-2 outperformed the other models across all metrics when tested with clean input text in most scenarios [27]. Predicting the first character proved to be the most challenging task for all transformer models, while the prediction of subsequent parts of words became progressively easier. Longer context windows generally improved prediction accuracy, though this advantage diminished when input noise was high. Overall, input noise significantly impacted performance, with Transformer-XL demonstrating the highest robustness to noise, while GPT and GPT-2 were more susceptible.

These findings suggest that LLMs, particularly more advanced implementations like GPT-3 and GPT-4, hold substantial promise for enhancing BCI-based communication systems. Their ability to leverage broader semantic and syntactic context positions LLMs as powerful tools for predictive text generation in noisy and constrained BCI settings.

## 4. Discussion

Early studies proposing the use of simple language models for BCI spellers’ implementation suffered from several shortcomings, including reliance on finite state machines (simple character n-gram models), high sensitivity to noise, the necessity for pretraining, and the neglect of language context. These limitations were mainly attributable to the difficulty of integrating more sophisticated language models into BCI systems, as such models were often too computationally complex for real-time classification algorithms. By contrast, the rapid development of advanced language models, particularly modern LLMs, promises to substantially enhance the predictive performance of BCI systems (Figure 1).

LLMs can effectively provide word predictions during text composition by supporting a broad and high-level understanding and generation of natural language patterns in an unsupervised manner. They are highly proficient in performing a wide range of NLP tasks and can be made compact enough to integrate into BCI software. In particular, pretrained transformer-based autoregressive language models have demonstrated success in delivering fast and efficient character-level predictions. These predictions are generated by deep learning-based neural networks that automatically predict the next character based on prior input, while also capturing dependencies across longer sequences. Autoregressive transformer models such as GPT, which leverage parallelization and benefit from pre-training and fine-tuning, offer a significant improvement over previous systems that relied on simpler language models.

Among these, GPT-2 has shown considerable promise as a candidate for integration into BCI systems, although testing has so far been limited to simulated conversations rather than real-world BCI scenarios [71]. GPT and GPT-2 models, which use subword tokenization and beam search, perform well on clean input data but are particularly vulnerable to character-level typing errors in the input history. To address this limitation, training models on noisy data may reduce susceptibility to input noise and improve the generalizability of language models [64]. Prediction accuracy can also improve with longer texts and dialogues, as transformer-based models are particularly effective at capturing and utilizing long-range contextual information.

LLMs, such as GPT-3.5, GPT-4, and Llama2 (Large Language Model Meta AI), are also capable of processing more complex aspects related to natural language understanding. These include sentiment analysis (or opinion mining—the process of determining the emotional tone behind a body of text, which involves classifying input into categories such as positive, negative, or neutral, and can be refined to detect specific emotions like joy, anger, sadness, or sarcasm), text classification, natural language inference, and semantic understanding [50].

In the realm of natural language generation, LLMs also demonstrate impressive capabilities, including summarization, dialogue generation, machine translation, question answering, and a wide range of open-ended generation tasks.

Moreover, BCI systems may benefit from the integration of Large Concept Models (LCMs), which are AI models that incorporate structured conceptual knowledge alongside textual information [72]. Concept-Centric Learning allows LCMs to learn abstract concepts and relationships between them instead of only processing word sequences. By leveraging structured knowledge sources (e.g., knowledge graphs, ontologies), LCMs can perform logical reasoning and inference that go beyond statistical pattern matching. As a result, it is conceivable that future BCI systems powered by advanced language models could support more complex and nuanced communicative interactions.

However, full integration of LLMs with BCI systems for patient communication presents a number of challenges and potential limitations. These include the need for real-time computational efficiency, the reliability of communication outputs, patient compliance, and significant ethical considerations—all of which must be carefully addressed.

### 4.1. Key Challenges for the Integration of LLMs with BCI Spellers

The integration of LLMs into BCI spellers introduces several critical issues regarding runtime performance, resource efficiency, and system scalability. Effective deployment demands careful evaluation of inference speed, computational cost, and infrastructure requirements to ensure practical and reliable operation.

LLMs, particularly state-of-the-art models such as GPT-4, are computationally intensive, requiring substantial memory, storage, and processing power—often necessitating dedicated hardware such as GPUs (Graphics Processing Units), TPUs (Tensor Processing Units), or specialized AI accelerators. As a result, real-time integration within BCIs typically relies on cloud-based APIs, where efficient model deployment hinges on balancing speed, accuracy, and resource consumption.

Several optimization strategies have been developed to address these challenges. Techniques such as quantization (reducing the precision of the numerical values used in the model, from higher-precision formats to lower-precision formats), model pruning (removing redundant or less important parts of a neural network), and caching can significantly reduce model size and latency without substantially compromising performance.

Quantization is one of the most impactful developments for LLMs, enabling inference on consumer-grade GPUs, mobile devices, and offline environments. However, quantization is not lossless—models may show degraded reasoning, especially on long-context tasks. Model pruning is also a promising strategy for scaling LLM deployment, particularly when paired with quantization and distillation, as it offers a way to compress models efficiently while retaining much of their capability. However, although model pruning offers clear advantages in reducing model size and computational cost, it is not widely adopted in LLMs because most current inference frameworks and hardware accelerators do not yet efficiently support sparse matrix computations, which are essential for realizing the performance gains from pruning.

Moreover, cloud-based dynamic scaling enables resource-efficient operation, particularly in real-time environments such as BCI-based communication systems.

A promising alternative to full-scale LLMs involves the use of smaller, task-optimized variants, such as Meta’s LLaMA, GPT-derived lightweight models, and Mistral AI architectures. These models are engineered for high performance under limited computational constraints, making them well-suited for edge devices and fine-tuned, domain-specific applications. Model distillation, where a compact model is trained to replicate the behavior of a larger one, further enhances efficiency by enabling speculative decoding (precomputing likely tokens), reducing latency, and optimizing energy usage.

Many lightweight LLMs (e.g., based on LLaMA, Mistral) are available as open-source models, allowing researchers and developers unrestricted access to their architecture, training data, and parameters. This openness fosters customization and facilitates integration into diverse BCI applications without reliance on proprietary platforms.

Despite these advances, a persistent limitation in the deployment of LLMs lies in their limited interpretability. Unlike rule-based systems, LLMs exhibit non-deterministic behavior, with identical inputs potentially yielding different outputs due to stochastic sampling. They lack transparent reasoning pathways, and their complex parameterization complicates efforts to trace specific outputs to underlying mechanisms. Although fine-tuning LLMs on domain-specific datasets can improve performance, determining which layers or weights require modification remains a significant challenge.

To enable efficient real-time adaptation within BCI systems, recent methods such as LoRA (Low-Rank Adaptation of LLMs) [73] and Adapter modules [74] offer lightweight, modular alternatives for fine-tuning without the need to retrain entire models. These approaches hold particular promise for adaptive BCI environments that demand responsiveness and personalization.

Despite all the latest progress on fine-tuning LLMs, retrieval augmentation, and test-time training [75], perhaps one of the most critical issues in the field is the limited capacity of deep learning models for continual learning [76,77], a requirement central to dynamic, long-term use in BCI applications. Current models are susceptible to catastrophic forgetting [78], wherein previously acquired knowledge is rapidly overwritten when the model is exposed to new data. While techniques such as LoRA, Adapters, and Retrieval-Augmented Generation (RAG) [79] can partially mitigate this problem, a comprehensive solution remains elusive.

Emerging methods such as experience replay or rehearsal-based learning, which revisit previously encountered instances during new learning, may support the retention of learned representations over time [80]. Complementary strategies, including Born-Again Neural Networks (BANNs) [81], a neural network training method based on self-distillation where a large model (teacher) is used to train a smaller model (student), also show some potential to address this challenge. For instance, the transfer of useful soft knowledge between generations might stabilize past knowledge and then mitigate catastrophic forgetting.

Ultimately, the selection of an appropriate LLM for BCI integration must reflect a careful trade-off between model complexity, interpretability, computational requirements, and deployment context (see Table 2). In summary, while substantial challenges remain—particularly in real-time deployment and lifelong learning—LLMs continue to offer transformative potential for enhancing BCI-based communication systems. Ongoing advancements in model optimization, fine-tuning, and continual learning are expected to play a pivotal role in realizing this integration at scale.

**Table 2:** Comparative overview of common LLMs based on key attributes such as Training Data Size, Feature Engineering, Model Complexity, Interpretability, Performance, and Hardware Requirements.

Model	Training Data Size ^1^	Feature Engineering ^2^	Model Complexity ^3^	Interpretability ^4^	Performance ^5^	Hardware Requirements ^6^
**GPT-3**	~570 GB of text data	Minimal manual feature engineering; relies on extensive unsupervised learning	175 billion parameters	Low; operates as a black-box model	High performance across diverse NLP tasks	Requires substantial computational resources for training and inference
**GPT-2**	~40 GB of text data	Minimal manual feature engineering; utilizes unsupervised learning	1.5 billion parameters	Low; similar black-box characteristics as GPT-3	Competent performance on various NLP tasks, though less capable than GPT-3	Moderate hardware requirements; more accessible than GPT-3
**BERT**	Trained on 16 GB of text data	Incorporates tokenization and context handling; designed for bidirectional context understanding	340 million parameters	Moderate; allows for some interpretability through attention mechanisms	Excels in tasks requiring an understanding of context within sentences	Lower hardware requirements; feasible for deployment on consumer-grade GPUs
**RoBERTa**	Trained on 160 GB of text data	Builds upon BERT with optimized training approaches and larger data volume	355 million parameters	Moderate; retains interpretability features similar to BERT	Outperforms BERT on several NLP benchmarks due to enhanced training	Requires more computational power than BERT but remains manageable
**T5**	Trained on 120 TB of text data	Treats all NLP tasks as text-to-text transformations; requires task-specific input formatting	11 billion parameters	Low; complexity increases with model size, reducing transparency	High versatility across a wide range of NLP tasks	Demands significant computational resources, though less than GPT-3
**XLNet**	Trained on billions of words	Integrates permutation-based training to capture bidirectional contexts	340 million parameters	Moderate; attention mechanisms provide some level of interpretability	Achieves strong performance on tasks involving contextual understanding	Comparable hardware requirements to BERT and RoBERTa
**Llama 2**	Trained on 2 trillion tokens	Utilizes advanced training techniques with a focus on efficiency	Model sizes up to 65 billion parameters	Low; large-scale models with limited transparency	Demonstrates robust performance across various applications	High hardware demands, though optimized for better efficiency than some counterparts
**Llama 3**	Trained on up to 15 trillion tokens	Incorporates extensive pre-training and human fine-tuning	Model sizes up to 405 billion parameters	Low; complexity and scale limit interpretability	Superior performance, handling complex tasks, and supporting multiple languages	Exponentially higher hardware and training intensity compared to Llama 2
**DeepSeek R1**	Not specified	Employs reinforcement learning and a “mixture of experts” approach	671 billion parameters, with selective activation reducing active parameter count to 37 billion for each token	Moderate; the “mixture of experts” method may offer enhanced interpretability	Recognized for superior performance in tasks like math and coding	Reduced power and processing needs; operates effectively on less advanced hardware

^1^**Training Data Size** refers to the volume of text data used during the model’s training phase. Larger datasets can enhance model performance but also require more computational resources. ^2^ **Feature Engineering** describes the extent of manual input required to design features for the model. Many modern LLMs minimize manual feature engineering, relying instead on unsupervised learning techniques. ^3^ **Model Complexity** indicates the number of parameters within the model. Higher parameter counts can lead to improved performance but also increase computational demands and reduce interpretability. ^4^ **Interpretability** assesses how easily humans can understand and trace the model’s decision-making processes. Larger, more complex models often function as “black boxes”, making interpretability challenging. ^5^ **Performance** reflects the model’s effectiveness across various Natural Language Processing (NLP) tasks, including text generation, translation, and comprehension (evaluated on Massive Multitask Language Understanding—MMLU—[82], HellaSwag, Winogrande, and related generative benchmarks as reported in official technical reports). ^6^ **Hardware Requirements** denote the computational resources necessary for training and deploying the model. Larger models typically require more advanced hardware setups.

In predictive communication tasks, each model exhibits distinct advantages in terms of accuracy, robustness, and efficiency (see Table 3). For instance, GPT-4 and LLaMA 3 demonstrate superior performance in tasks requiring sophisticated generative capabilities, while models such as BERT and RoBERTa excel in deep contextual understanding. DeepSeek R1 offers a balanced profile across multiple evaluation criteria, making it a viable candidate for general-purpose applications. Nevertheless, for predictive writing applications, GPT-4, T5, and Llama models all offer high efficiency.

**Table 3:** **Comparative evaluation of prominent LLMs based on accuracy, calibration, robustness, and efficiency in predictive writing (0–10 Scale)**.

Model *	Accuracy ^1^	Calibration ^2^	Robustness ^3^	Efficiency ^4^
**GPT-4**	10	8	10	3
**GPT-3**	8	6	8	3
**BERT**	6	8	8	7
**RoBERTa**	7	8	9	6
**T5**	8	8	6	6
**Llama 2**	8	6	8	8
**Llama 3**	10	8	10	8
**DeepSeek R1**	8	6	6	10

^1^**Accuracy** describes the model’s ability to generate correct and relevant responses across various tasks (evaluated on Massive Multitask Language Understanding—MMLU—[82], HellaSwag, Winogrande, and related generative benchmarks as reported in official technical reports). ^2^ **Calibration** corresponds to the alignment between the model’s confidence in its predictions and the actual correctness of those predictions (based on Expected Calibration Error, [83,84]). ^3^ **Robustness** indicates the model’s resilience to input variations, adversarial examples, and its performance consistency across different scenarios (based on AdversarialQA and BoolQ under perturbation, robustness in MMLU under noisy prompts, faithfulness in summarization tasks, resistance to prompt injection or misleading instruction). ^4^ **Efficiency** describes the model’s capability to assist users effectively in tasks like code completion, text autocompletion, and other predictive writing applications (based on FLOPs, latency, hardware footprint, and inference cost per token). For each category, published benchmark scores or performance stats are normalized across all models on a 0–10 scale, and consistency is verified with technical reports. * Synthesis. Accuracy: GPT-4 achieves state-of-the-art performance in predictive generation tasks (MMLU, HellaSwag, StoryCloze), outperforming previous GPT versions (OpenAI, 2023). GPT-3 is strong but less accurate than GPT-4, especially in long context reasoning. BERT and RoBERTa are not primarily generative; instead, they are masked language models (MLMs), which limits their predictive writing utility [85,86]. T5 is strong in sequence-to-sequence tasks and shows high performance in generative benchmarks. LLaMA 3 achieves GPT-4-comparable or better scores on many language modeling benchmarks [87]. DeepSeek R1 demonstrates high competence on MMLU and reasoning tasks with lower training compute [88]. **Calibration:** GPT-4 shows improved calibration compared to GPT-3 [83], though still overconfident in some cases. BERT/RoBERTa are well-calibrated in classification tasks [84] but not directly comparable in generative settings. T5 is moderately well-calibrated but exhibits overconfidence under distributional shifts. LLaMA 3 shows better calibration than LLaMA 2 and Open LLMs, but a little less than GPT-4. DeepSeek R1 lacks a published detailed calibration analysis but shows moderate uncertainty awareness in decoding. **Robustness:** GPT-4 is significantly more robust to adversarial perturbations, hallucinations, and input noise [89]. RoBERTa exhibits stronger adversarial robustness than BERT due to dynamic masking and larger data. T5 and LLaMA 3 show improved robustness under distributional shifts and perturbations. DeepSeek R1 is moderately robust, but its behavior under adversarial examples is less well-documented. **Efficiency:** GPT-4 and GPT-3 are resource-intensive; they are expensive to train and deploy. LLaMA 2/3 are much more efficient (especially in smaller variants) with near-GPT performance. DeepSeek R1 is highly efficient—strong performance with ~30× less compute than GPT-4 [88]. BERT and RoBERTa are efficient for smaller tasks but scale poorly for long-range generation. T5 is resource-intensive in full-size models but scalable across tiers (small to XXL).

#### 4.1.1. Communication Error Correction

LLMs are prone to generating incorrect, misleading, or implausible outputs that nonetheless appear coherent, a phenomenon commonly referred to as LLM hallucinations. These hallucinations stem from the fact that LLMs generate text based on statistical patterns in training data, without a true understanding of meaning or context. Contributing factors include limitations in training data, biases, constraints on context-window size, and the autoregressive generation process, in which each token is predicted sequentially, sometimes drifting into logically inconsistent or false outputs. In the context of BCI-based communication, where speed, accuracy, and reliability are critical, mitigating such hallucinations is essential. One promising strategy is the use of Retrieval-Augmented Generation (RAG), which enhances LLM outputs by integrating external knowledge bases during generation. This technique can significantly reduce hallucinations and improve the factual accuracy of the model’s suggestions, ultimately boosting communication reliability.

Traditionally, error correction in BCI speller systems has relied on manual user intervention—requiring the user to repeat character selection steps or activate backspace commands—resulting in increased typing time and reduced overall performance [90]. To streamline this process, alternative approaches have proposed maintaining multiple candidate characters for each selection, enabling users to choose from a set of suggestions, thereby reducing error correction time and cognitive load [65]. In addition to these approaches, some EEG-based BCI systems have incorporated automatic error correction based on error-related potentials (ErrPs)—neural responses that reflect the user’s perception of an error [91,92,93]. ErrPs typically include a negative deflection (error-related negativity) followed by a positive component, thought to correspond to conscious error recognition. In some cases, additional error-related signals may precede the execution of an incorrect action. The integration of ErrPs into BCI classification pipelines can indeed contribute to enhancing both speed and accuracy of character selection [94].

Beyond general error signals, language-specific event-related potentials (ERPs) such as the N400 and P600, typically elicited by semantically or syntactically incongruent linguistic inputs, may provide additional insight into whether a selected word or letter fits the contextual meaning [95,96]. These signals offer a neurophysiological means of verifying contextual correctness in BCI-mediated language production. Importantly, LLMs also offer the potential for automatic, context-aware error correction without requiring explicit user input. For example, a model may autonomously detect and correct spelling or grammar errors based on contextual cues in the evolving text stream. Nonetheless, ErrPs could still serve a complementary role, triggering automated correction mechanisms when an error is detected, or confirming corrections based on neural signatures of recognition. Furthermore, error correction need not be limited to individual characters. Most BCI language models currently focus on next-character prediction; nevertheless, there is significant potential to extend these systems toward complete word or sentence prediction. As LLMs excel at word-level and phrase-level predictions, BCI systems could be designed to generate candidate words or short sequences after only a few characters have been typed. This would substantially increase communication speed and reduce cognitive effort.

Looking ahead, the most advanced integration of LLMs and BCIs may involve neuroadaptive interfaces, wherein communication occurs implicitly. In such systems, the acceptance or rejection of predicted outputs could be guided by decoding anticipatory brain signals that reflect user intent—enabling seamless, subconscious interaction with the model [97]. This vision points toward a future in which LLM-BCI systems are not only faster and more accurate but also capable of aligning implicitly with the user’s cognitive and communicative goals.

#### 4.1.2. Patient-Centered Communication Perspective

Modern LLMs support flexible text generation beyond fixed linguistic patterns found in general corpora and have demonstrated strong performance in context-specific tasks, including patient-specific communication. By leveraging syntactic and semantic features of language along with contextual cues, and potentially integrating complementary bio-signals or behavioral data, LLMs can significantly enhance BCI-mediated text composition, even enabling the selection of complete sentences.

In addition, by leveraging the sentiment analysis feature of LLMs [50], novel BCI spellers could facilitate expressive communication by simulating prosody and emotional nuance, enabling more embodied interactions for individuals with severe motor impairments [8,11,12,13,98]. To evaluate such systems, traditional autoregressive model metrics may be supplemented or adapted to assess semantic and emotional alignment, for example, through semantic-sentiment perplexity or accuracy.

Importantly, to ensure personalized and proper communication through BCI spellers, user-specific fine-tuning and filtering can align outputs with the individual’s vocabulary, communication style, and values. Privacy remains a critical consideration, especially when LLMs are accessed via APIs or deployed in cloud environments using sensitive personal or neural data. In these cases, data encryption of inputs and outputs is essential to protect user confidentiality. Alternatively, deploying LLMs locally—while more hardware-intensive—offers a robust approach to maintain data privacy and autonomy.

#### 4.1.3. LLMs for Brain Decoding in BCI Spellers

LLMs also hold the potential to advance neural decoding in BCI spellers by serving as foundational models trained to understand neuroscientific data. For instance, a pilot model, Neuro-GPT, combining an EEG encoder with a GPT model, trained using a self-supervised task that can extract inherent and relevant features of EEG segments, showed improved classification performance over models trained from scratch [99]. Similarly, a fine-tuned GPT-3.5 Turbo model, prompted with preprocessed intracranial EEG signals categorized by frequency bands and brain regions, successfully generated interpretable outputs describing neural activity in specific cognitive states [71].

In a further recent study, a Thought2Text system demonstrated the feasibility of directly translating EEG signals into textual outputs [100]. Initially, EEG features associated with visual processing were extracted, and several LLMs (LLaMA-v3, Mistral-v0.3, Qwen2.5) were fine-tuned on multimodal data (images, text, EEG). These models were then evaluated using standard natural language generation (NLG) metrics such as BLEU, METEOR, ROUGE [101], BERTScore [102], as well as GPT-4-based evaluations of fluency (for grammar) and adequacy (for accuracy in conveying meaning). Preliminary results confirmed that all multimodally fine-tuned LLMs could effectively translate visual EEG stimuli into coherent text, performing significantly above chance in most of the metrics.

For real-time neural decoding, deep learning-based EEG classification methods [1] can outperform traditional algorithms, particularly in discriminating visual evoked potentials (VEPs). These approaches benefit from automatic feature extraction, end-to-end training, and generalization across time and subjects despite high variability. For example, predictive modeling of stimulus-evoked EEG patterns has been used to enhance classification efficiency [19,103]. In addition, transfer learning has proven effective in addressing mismatched data distributions, offering a critical advantage over some conventional machine learning techniques [104]. Overall, the integration of LLMs into neural decoding pipelines of BCI spellers holds substantial promise to boost both accuracy and speed in communication.

### 4.2. Other LLM-BCI Applications

Beyond communication support for individuals with severe motor impairments, LLM-enhanced BCI systems may also benefit those with developmental learning disorders, such as dyslexia or dysgraphia. These systems can support rapid, accurate text generation by reinforcing visuolinguistic processing, thus functioning both as assistive tools and as intervention technologies.

In parallel to their success in motor rehabilitation, BCI/BMI platforms may promote localized neuroplasticity within the visuolinguistic network, as observed previously in the sensorimotor domain [105,106,107,108]. Within this perspective, recent proof-of-concept studies suggested novel BCI applications in language recovery, showing potential improvements in language production and partial restoration of function in aphasic patients following stroke [109,110].

BCIs are primarily designed as an alternative means of interpreting the intention of individuals with severe neurological disorders. However, the rapid development of LLM-integrated BCI may open up promising new applications for healthy individuals as well. LLM-based neurotechnology may expand applications to include healthy individuals. Potential use cases include HCI, augmented communication interfaces, and executive control of external systems [28]. In these domains, LLM-enhanced BCIs could provide more intuitive, context-aware, and adaptive interactions, further bridging the gap between brain activity and intelligent systems.

## 5. Conclusions

Significant advancements have been made since the seminal study that first demonstrated BCI-based communication in ALS patients [111]. The remarkable development of AI-based language models, such as LLMs, now offers a radical breakthrough for transforming BCI-mediated communication. However, optimizing LLM performance in this context requires a careful balance between speed, accuracy, computational cost, and scalability. Additionally, the implementation of patient-centered BCI systems must address critical factors such as usability, user compliance, and ethical considerations [112], which should be thoroughly explored in future research. Furthermore, the integration of LLMs into neural decoding still presents several challenges, including constraints related to real-time processing, limitations in training data, and variability in individual neural responses.

To date, LLM-enhanced neural decoding approaches have not yet been applied to BCI systems aiming at direct speech decoding from EEG activity. Nevertheless, ongoing advancements in AI, deep learning methodologies, and neural signal processing provide evidence that speech decoding from non-invasive EEG signals may, to some extent, be attainable [14,15,16]. These promising findings might, in the future, parallel the recent advancements achieved with invasive BCI technologies [9]. Importantly, non-invasive systems remain more practical, versatile, and safer for clinical use compared to invasive alternatives. Advances in the integration of LLMs with real-time neural decoding would likely permit to move progressively closer to naturalistic brain-to-speech communication. The eventual convergence of AI and BCI technologies would thus represent a substantial breakthrough, enabling fast, efficient, and user-adaptive neurotechnologies with broad translational potential.

## Figures and Tables

**Table 1:** Key differences between autoregressive and transformer-based models. Notes: GPT (like GPT-4) is both autoregressive and transformer-based because it uses the transformer architecture but generates text token-by-token. BERT is purely transformer-based but not autoregressive because it processes input bidirectionally.

Feature	Autoregressive Models	Transformer-Based Models
**Processing**	Sequential (one token at a time)	Parallel (whole sequence at once)
**Speed**	Slow (token-by-token)	Fast (parallel computation)
**Architecture**	RNNs, LSTMs, AR processes	Self-attention, multi-head attention
**Context**	Limited to past tokens (causal)	Can use full context (self-attention)
**Examples**	GPT (autoregressive), ARIMA, LSTMs	GPT, BERT, T5, full transformer

## Data Availability

Not applicable.
